# COVID-19 excess mortality among long-term care residents in Ontario, Canada

**DOI:** 10.1371/journal.pone.0262807

**Published:** 2022-01-20

**Authors:** Noori Akhtar-Danesh, Andrea Baumann, Mary Crea-Arsenio, Valentina Antonipillai

**Affiliations:** 1 School of Nursing, McMaster University, Hamilton, Ontario, Canada; 2 Department of Health Research Methods, Evidence, and Impact, McMaster University, Hamilton, Ontario, Canada; 3 Global Health, McMaster University, Hamilton, Ontario, Canada; Universiti Teknologi Malaysia - Main Campus Skudai: Universiti Teknologi Malaysia, MALAYSIA

## Abstract

The coronavirus disease 2019 (COVID-19) has had devastating consequences worldwide, including a spike in global mortality. Residents of long-term care homes have been disproportionately affected. We conducted a retrospective cohort study to determine the scale of pandemic-related deaths of long-term care residents in the province of Ontario, Canada, and to estimate excess mortality due to a positive COVID-19 test adjusted for demographics and regional variations. Crude mortality rates for 2019 and 2020 were compared, as were predictors of mortality among residents with positive and negative tests from March 2020 to December 2020. We found the crude mortality rates were higher from April 2020 to June 2020 and from November 2020 to December 2020, corresponding to Wave 1 and Wave 2 of the pandemic in Ontario. There were also substantial increases in mortality among residents with a positive COVID-19 test. The significant differences in excess mortality observed in relation to long-term care home ownership category and geographic region may indicate gaps in the healthcare system that warrant attention from policymakers. Further investigation is needed to identify the most relevant factors in explaining these differences.

## Introduction

The coronavirus disease 2019 (COVID-19) has had devastating consequences worldwide, including a spike in global mortality. One group that has been disproportionately affected are residents of long-term care (LTC) homes. Compared with other Organisation for Economic Co-operation and Development countries, Canada had a relatively low overall COVID-19 mortality rate but the highest proportion of LTC deaths. Residents of LTC homes in Canada represented 81% of all reported COVID-19 deaths compared with an average of 38% in other countries [1].

Globally, countries have reported a significant number of COVID-related deaths in LTC settings. In Sweden, more than 50% of all deaths occurred in LTC homes, the highest among the Scandinavian countries [2]. In Italy, municipalities with care homes had higher rates of excess deaths related to COVID compared to those without a care home [3]. Excess deaths were estimated 7% in all care homes in England during the first 23 weeks of COVID-19; of those, 65% of all deaths were attributable to the virus [4]. Similarly, in Belgium residents of care homes were 130 times more likely to die from a COVID-19 infection than the rest of the population [5]. Finally, in a recent Canadian study of excess mortality, it was found that COVID-19 deaths in long-term care were approximately 50% greater than reported COVID-19 fatalities in Italy, Spain, the United Kingdom and United States [4]. Across Canadian regions, the percentage of COVID-19 fatalities reported in LTC has remained close to double the OECD average throughout the COVID-19 pandemic [4].

In the light of the impact of the pandemic on residents of long-term care homes, further investigation into excess mortality is required. Mortality is one indicator of how sectors such as LTC were able to protect their residents during the most severe stages of the pandemic. Using resident-level data from linked health administrative databases, this paper provides an analysis of the scale of COVID-19 deaths among LTC residents to further assess the extent of excess mortality from infection. The paper focuses on Canada’s most populous province of Ontario and one of the hardest hit during the pandemic [6].

There are 626 LTC homes in Ontario with approximately 78,000 residents at any one time [1]. Ownership, operation, and providers differ. Currently, 57% of the homes are for-profit and are privately owned, 27% are not-for-profit, and 16% are public and operated by municipalities [7]. From January 15, 2020, to February 28, 2021, there were 14,960 cases of COVID-19 among LTC residents in Ontario and 3871 deaths [8]. This represents 64.5% of all pandemic-related deaths in the province [9].

The incidence rate for COVID-19 deaths among LTC residents in Ontario was 13 times higher than the rate for adults 69 years of age or older living in the community [10]. Evidence demonstrates that for-profit LTC homes have lower levels of staffing, more complaints from residents and family, more acute care hospital admissions, and higher mortality rates [11, 12]. Lack of preparation, shortage of staff, and overcrowding have contributed to excess mortality in the LTC sector, and mortality increases with age and pre-existing medical conditions [13–16]. Furthermore, personal protective equipment, reduced use of part-time staff, and early implementation of infection protocols have been linked to lower rates of mortality in nursing homes [17].

We conducted a retrospective cohort study with the aim of understanding the scale of COVID-19 deaths among LTC residents in Ontario and estimating excess mortality due to a positive COVID-19 test adjusted for demographics and regional variations. For the purposes of the study, excess mortality was defined as an estimate of the additional number of deaths within 30 days of a COVID-19 test compared to the expected number of deaths [18]. It is a measure encompasses all causes of death, directly or indirectly associated with a positive COVID-19 test, and provides a metric of the overall mortality impact of the pandemic [19]. We found significant differences in excess mortality with regard to ownership category and geographic region of LTC homes. This may indicate gaps in the healthcare system that warrant attention from policymakers.

## Materials and methods

### Data sources

The study focused on LTC residents in Ontario from January 2019 to December 2020 and used population-based health administrative data sets from the Institute for Clinical Evaluative Sciences (ICES) and the Resident Assessment Instrument—Minimum Data Set (RAI-MDS) 2.0. The RAI-MDS contains information on resident clinical, functional, and psychosocial characteristics. The study obtained LTC home admission and discharge dates from the Continuing Care Reporting System (CCRS). COVID-19 testing and positivity data were obtained from linking these health administrative databases to the Ontario Laboratories Information System (OLIS), which acquires SARS-CoV-2 infection test data from hospitals, commercial laboratories, provincial public health laboratory and COVID-19 assessment centers. Mortality and demographic data were obtained through the Registered Persons Database (RPDB). The data sets were linked via unique encoded identifiers.

The age groups for residents were coded as 65–69, 70–74, 75–79, 80–84, and ≥85 years. The following variables were included in the analysis for each resident: result of last COVID-19 test (positive or negative from March 2020); region of Ontario (Central, Southwest, East, North); income quintile based on postal code of the LTC home; frailty index (classified as robust, pre-frail, frail); ownership category of LTC home (municipal, non-profit, private); and Changes in Health, End-stage disease Symptoms and Signs Scales (CHESS) comorbidity score [20, 21]. The income quintile was coded as 1 (lowest income level) to 5 (highest income level). The CHESS score varies from 0 (no instability in health) to 5 (high unstable health).

The study was conducted in accordance with the Declaration of Helsinki. The study was approved by authors’ institutional ethics board. ICES is a prescribed entity under section 45 of Ontario’s Personal Health Information Protection Act. Section 45 authorizes ICES to collect personal health information, without consent, for the purpose of analysis or compiling statistical information with respect to the management of, evaluation or monitoring of, the allocation of resources to or planning for all or part of the health system. Projects conducted under section 45, by definition, do not require informed consent. This project was conducted under section 45 and approved by ICES’ Privacy and Legal Office.

### Statistical analysis

Summary statistics were reported as mean and standard deviation (SD) for continuous variables and as frequency and percentage for categorical variables and were compared using t-tests and chi-squared tests, respectively. We compared crude mortality rate per 1000 person-months between 2019 and 2020. We first calculated the crude mortality rate per month from January 2019 to December 2020, where rate for each month in 2020 can be compared with the rate of same month in 2019. A flexible parametric model [22, 23] was used to estimate mortality rates based on COVID-19 test results from March 2020 to December 2020. The model provides smooth estimates of survival and mortality using restricted cubic splines on the log cumulative excess hazard scale. Compared to standard survival models such as Cox regression and parametric survival models, it adopts a piecewise approach and is more flexible in mimicking the actual trends in mortality (hazard rate [HR]) and survival patterns [24].

We fitted the model by incorporating age group, sex, region, income quintile, frailty index, CHESS score, ownership category, and interaction term between each two variables into a multiple statistical model using a forward approach. We used the likelihood ratio test to compare different models toward reaching a final model. All variables included in the final model were statistically significant. Statistical significance was evaluated with 2-sided p-values at a type I error rate of 0.05. Based on the final model, mortality was estimated for residents with positive or negative COVID-19 tests per 1000 person-days for over 30 days after each test while adjusting for the other variables in the model. The flexible parametric model was fitted using the freely available stpm2 program for Stata package [22]. All statistical analyses were conducted using Stata/MP 15.1 (Stata Corporation, College Station, TX).

## Results

**Fig 1:**
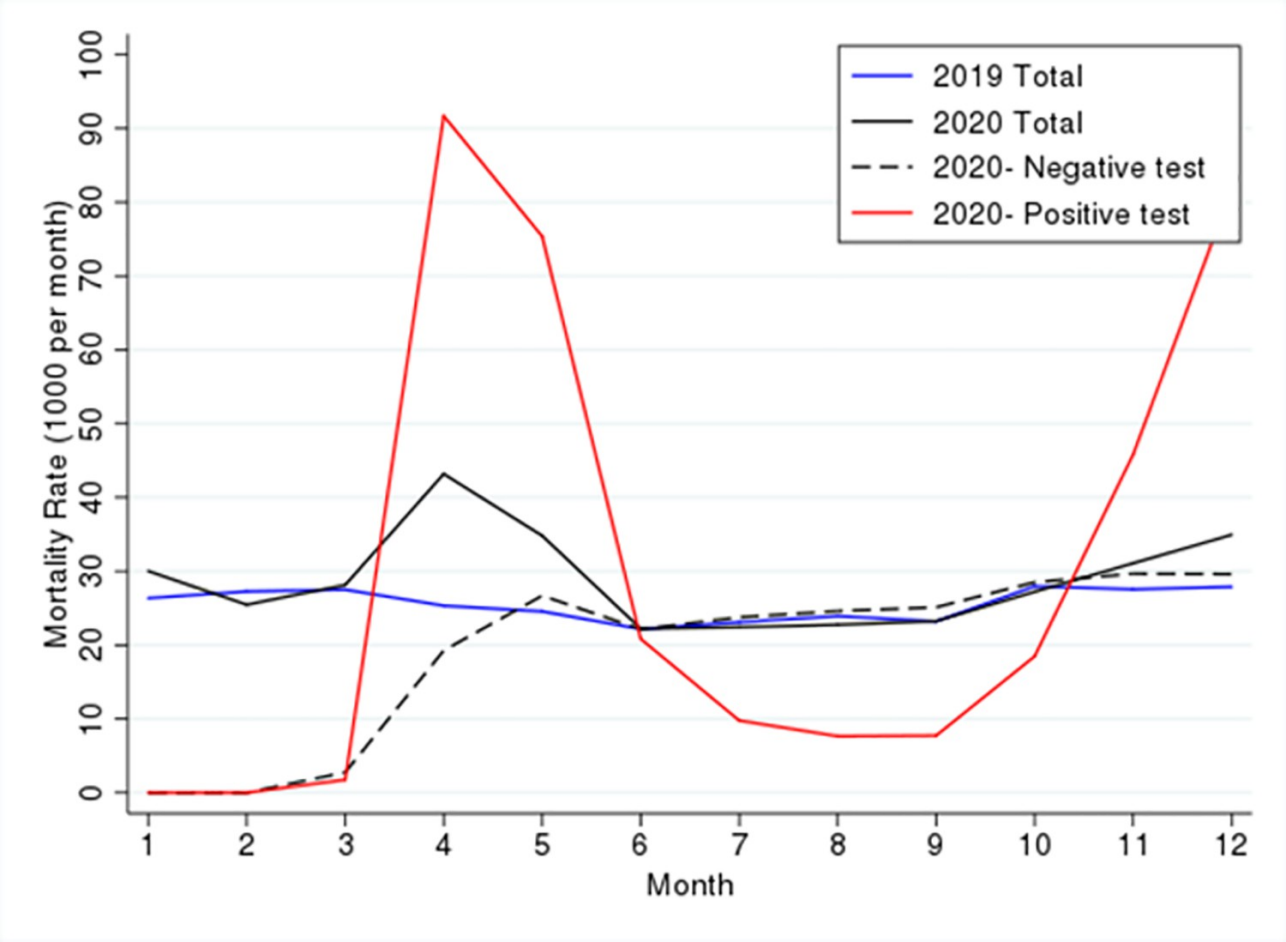
Crude mortality rate based on year and COVID-19 test result.

The study included a total of 120, 286 residents across the two years. In 2019 there were 102,897 LTC residents and in 2020 there were 89,034 LTC residents, of which 71, 645 were also residents in 2019. The crude mortality rates are depicted in Fig 1; compared to the same periods in 2019, crude mortality rate were higher from April 2020 to June 2020 and from November 2020 to December 2020, corresponding to Wave 1 and Wave 2 of the pandemic in Ontario.

Comparison of mortality rates from March 2020 to December 2020 between residents with positive and negative COVID-19 tests showed the rates for residents with negative tests were at the same level as in 2019, but there was significant variation for those with positive tests. The mortality rate increased sharply in April and May, decreased in June to September, and increased again in October to December. The spikes in mortality correspond to Wave 1 and Wave 2 of the pandemic.

Table 1 presents the number and percentage of residents between March 2020 and December 2020 based on their COVID-19 test results and demographic variables. Although the COVID-19 positivity rate was statistically significant for every variable, there were substantial differences between categories for some variables such as region and LTC home ownership. For example, the positivity rate was 23.4% for Central Ontario compared to only 1.8% for Northern Ontario. In addition, it was 5.4% for municipally owned homes compared to 16.6% for privately owned for-profit homes.

**Table 1:** Distribution, n (%), of COVID-19 tests based on demographics from March 2020 to December 2020.

Category	Negative Test	Positive Test	Total	P-value
**Sex**				
Male	20,586 (87.0)	3082 (13.0)	23,668	0.002
Female	45,150 (87.8)	6282 (12.2)	51,432	-
**Age group**				
65–69 years	3679 (86.4)	579 (13.6)	4285	0.001
70–74 years	5689 (86.7)	872 (13.3)	6561	-
75–79 years	8413 (87.4)	1211 (12.6)	9624	-
80–84 years	12,476 (87.1)	1844 (12.9)	14,320	-
≥85 years	35,479 (88.0)	4858 (12.0)	40,337	-
**Age:** Mean (SD)	84.3 (8.2)	83.8 (8.2)	84.3 (8.2)	<0.001
**CHESS**^a^ **scale:** Mean (SD)	0.72 (0.83)	0.60 (0.76)	0.71 (0.83)	<0.001
**Region**				
Central	15,662 (76.6)	4793 (23.4)	20,455	<0.001
Southwest	23,251 (92.3)	1932 (7.7)	25,183	-
East	17,492 (87.7)	2453 (12.3)	19,945	-
North	9287 (98.2)	174 (1.8)	9461	-
**Income quintile**				
1 (lowest)	19,946 (87.5)	2796 (12.5)	22,292	<0.001
2	13,917 (85.6)	2333 (14.4)	16,250	-
3	11,824 (87.6)	1677 (12.4)	13,501	-
4	10,940 (89.5)	1284 (10.5)	12,224	-
5 (highest)	9029 (88.4)	1183 (11.6)	10,212	-
**Frailty index**				
Robust	8518 (87.6)	1205 (12.4)	9723	<0.001
Pre-frail	21,666 (86.5)	3381 (13.5)	25,047	-
Frail	34,963 (88.1)	4737 (11.9)	39,700	-
**Long-term care home ownership**				
Municipal	14,822 (94.6)	850 (5.4)	15,672	<0.001
Non-profit	18,225 (90.0)	2027 (10.0)	20,252	-
Private	32,672 (83.4)	6487 (16.6)	39,159	-
**Total**	**65,736 (87.5)**	**9364 (12.5)**	**75,100**	-

^a^Changes in Health, End-stage disease and Symptoms and Signs.

In the following, a death was counted for a resident if it occurred within 30 days of a COVID-19 test. International studies suggest that most deaths caused by COVID-19 among LTC home residents occur within the first 28 to 30 days after a SARS-CoV-2 diagnosis [25, 26]. After 30 days, death among residents is more likely to be attributable to another disease or underlying condition [26].

Table 2 shows large differences in mortality based on COVID-19 test results and demographics. The maximum difference between the youngest and oldest groups of residents with a negative test was 2.3%, but it was 13.81% for those with a positive test. According to the frailty index, there was a 3.31% difference between the youngest and oldest groups of residents with a negative test and a 12.45% difference for those with a positive test. Also, there was a maximum difference of 1.50% between geographical regions (Central vs. North) among residents with negative tests, but the difference increased to 11.44% (East vs. North) among residents with positive tests.

**Table 2:** Mortality rate (%) based on COVID-19 tests and demographics for March 2020 to December 2020.

Category	Negative Test		Positive Test		Total	
	Mortality rate	P-value	Mortality rate	P-value	Mortality rate	P-value
**Sex**						
Male	5.76	<0.001	32.18	<0.001	9.13	<0.001
Female	4.18	-	21.27	-	6.24	-
**Age group**						
65–69 years	3.06	-	15.09	-	4.67	-
70–74 years	3.59	-	17.76	-	5.45	-
75–79 years	3.90	<0.001	19.62	<0.001	5.87	<0.001
80–84 years	4.22	-	23.94	-	6.71	-
≥85 years	5.36	-	28.90	-	8.14	-
**Region**						
Central	5.38	-	23.59	-	9.60	-
Southwest	4.44	<0.001	21.18	<0.001	5.67	<0.001
East	4.78	-	30.49	-	7.91	-
North	3.88	-	19.05	-	4.16	-
**Income quintile**						
1 (lowest)	4.69	-	24.51	-	7.14	-
2	4.81	-	26.35	-	7.88	-
3	4.41	0.098	25.34	0.244	6.96	<0.001
4	4.40	-	23.61	-	6.37	-
5 (highest)	5.11	-	23.36	-	7.17	-
**Frailty index**						
Robust	2.41	-	16.75	-	4.19	-
Pre-frail	3.74	<0.001	21.61	<0.001	6.12	<0.001
Frail	5.72	-	29.20	-	8.47	-
**Long-term care home ownership**						
Municipal	4.27	-	22.45	-	5.23	-
Non-profit	4.43	<0.001	23.48	0.044	6.32	<0.001
Private	5.00	-	25.58	-	8.36	-

In the following we compared overall mortality between positive and negative tests using a flexible parametric model while adjusting for the demographic and geographic factors. Therefore, the difference in mortality can be treated as the excess mortality between positive and negative tests. Potential predictors are shown in Table 3. The effects of each variable were adjusted for the other variables. There was an interaction between region of LTC home and COVID-19 test result. Using Central Ontario with negative test as the reference group, the highest risk of death for residents with positive tests was observed in Eastern Ontario (HR = 6.90; 95% CI: 6.24, 7.64), followed by Southwest Ontario (HR = 5.64; 95% CI: 5.00, 6.39), Central Ontario (HR = 5.27; 95% CI: 4.81, 5.78), and Northern Ontario (HR = 4.73; 95% CI: 3.32, 6.75).

**Table 3:** Predictors of death within 30 Days after COVID-19 test.

Variable	HR^a^ (95% CI)	P-value
Region, COVID-19 test result		
Central, negative	Reference group	-
Central, positive	5.27 (4.81, 5.78)	<0.001
Southwest, negative	0.77 (0.70, 0.84)	<0.001
Southwest, positive	5.64 (5.00, 6.39)	<0.001
East, negative	0.86 (0.78, 0.94)	0.002
East, positive	6.90 (6.24, 7.64)	<0.001
North, negative	0.73 (0.64, 0.82)	<0.001
North, positive	4.73 (3.32, 6.75)	<0.001
**+ test interaction with time**	1.06 (1.03, 1.08)	<0.001
**Sex**		
Male	Reference group	-
Female	0.58 (0.55, 0.62)	<0.001
**Age group**		
65–69 years	Reference group	-
70–74 years	1.16 (0.97, 1.38)	0.105
75–79 years	1.33 (1.12, 1.57)	0.001
80–84 years	1.53 (1.31, 1.79)	<0.001
≥85 years	2.00 (1.73, 2.32)	<0.001
**CHESS**^b^ **scale**	1.13 (1.09, 1.16)	<0.001
**Frailty index**		
Robust	Reference group	-
Pre-frail	1.44 (1.29, 1.61)	<0.001
Frail	2.06 (1.85, 2.29)	<0.001
**Long-term care home ownership**		
Municipal	Reference group	-
Non-profit	1.06 (0.97, 1.16)	0.235
Private	1.25 (1.16, 1.35)	<0.001

^a^Hazard rate.

^b^Changes in Health, End-stage disease and Symptoms and Signs.

The risk of death for COVID-19 positive patients was time dependent and increased daily (HR for interaction between a positive test and time = 1.06; 95% CI: 1.03–1.08). Risk of death in private LTC homes was 25% higher than in municipal homes (HR = 1.25, 95% CI: 1.16–1.35). There was no significant difference in risk of death between non-profit LTC homes and municipal homes (p = 0.235).

**Fig 2:**
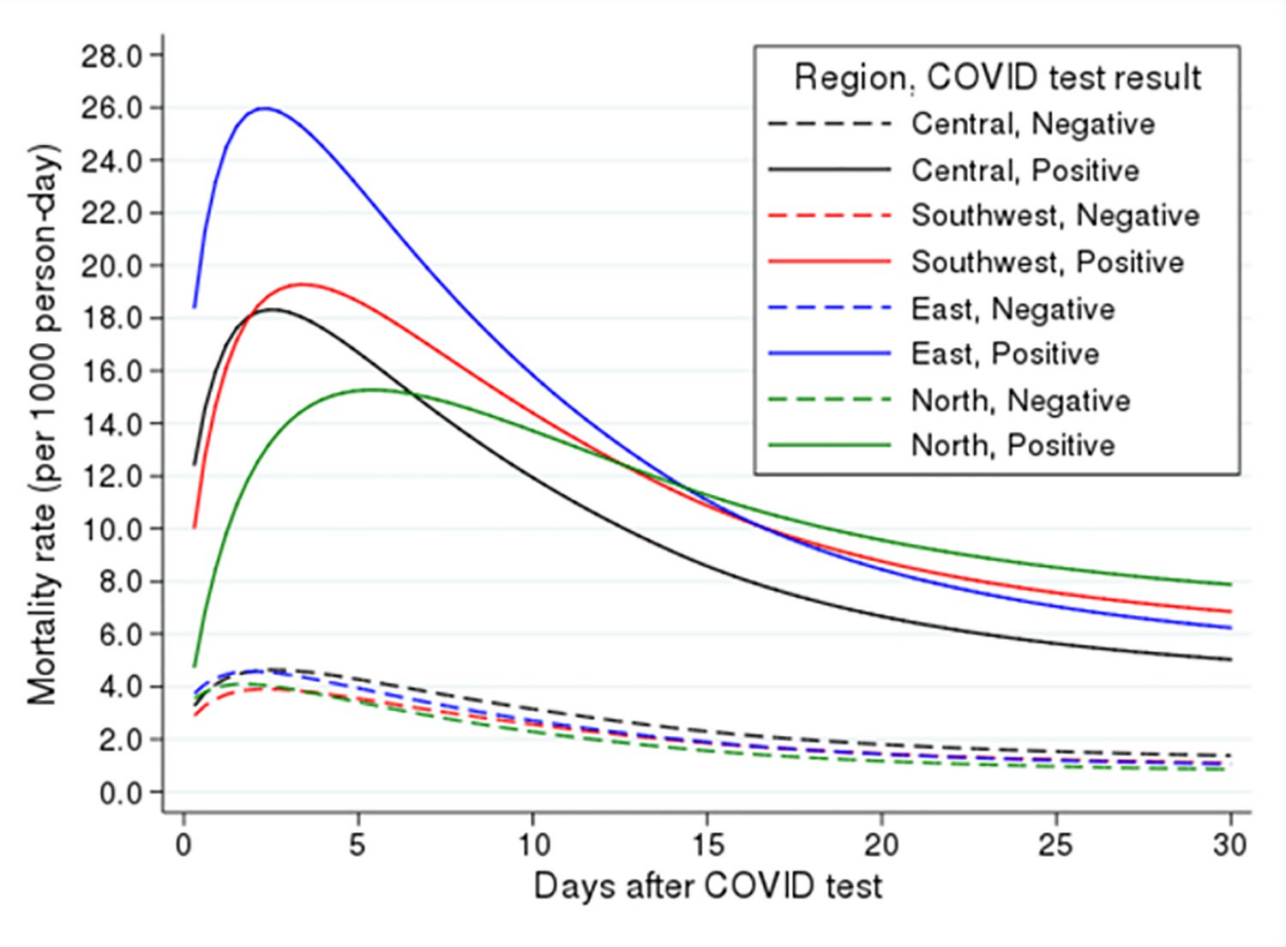
Adjusted mortality rate based on region and COVID-19 test result adjusted for sex, age group, Changes in Health, End-stage disease and Symptoms and Signs (CHESS) scale, frailty, and long-term care home ownership.

We also examined variation in mortality rate based on region and COVID-19 test result. Fig 2 shows the mortality rate (per 1000 person-day) for 30 days after a test. This figure is based on the final model and is adjusted for sex, age group, Changes in Health, End-stage disease and Symptoms and Signs (CHESS) scale, frailty, and long-term care home ownership.

**Fig 3:**
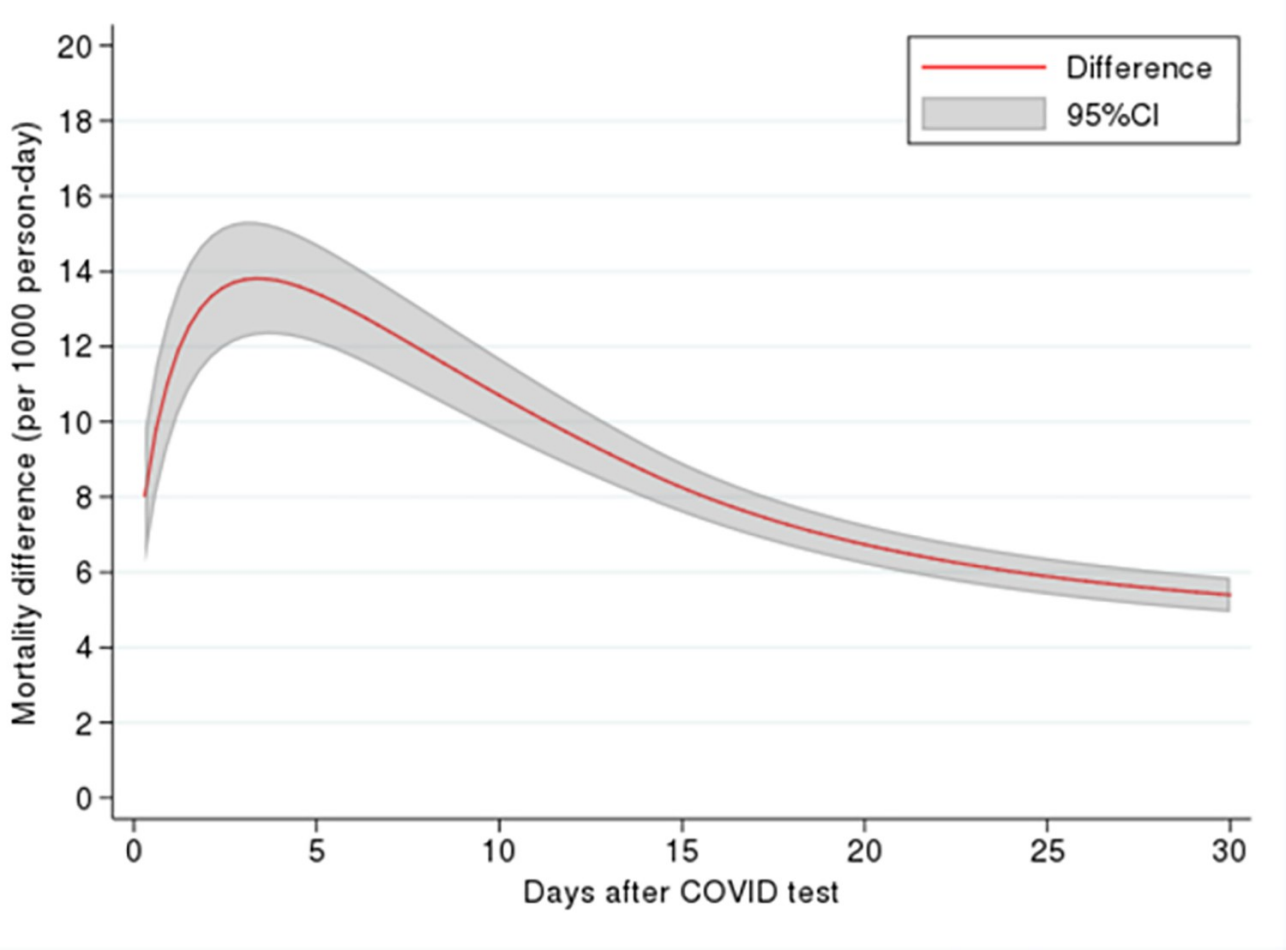
Excess mortality rate due to a positive COVID-19 test, adjusted for sex, age group, Changes in Health, End-stage disease and Symptoms and Signs (CHESS) scale, frailty, and long-term care home ownership.

Depending on the region, the mortality rate was at the highest level 3 to 5 days after a positive result and then subsided and plateaued after 20 days. We also estimated differences in mortality based on COVID-19 test as an approximate for excess mortality rate due to a positive test and found that excess mortality for a positive test started at 8 per 1000 person-days and increased sharply up to 4 days after the test (Fig 3). It then gradually decreased but remained elevated until 30 days after the test. The highest excess mortality was approximately 14 per 1000 person-days.

## Discussion

In this study, we compared crude mortality rates between 2019 and 2020 for LTC residents in Ontario. We also compared mortality rates between LTC residents with positive and negative COVID-19 tests. We chose to focus on deaths among LTC residents in Ontario as the rate of mortality was 13 times higher than the rate for older adults living in the community [10]. The difference in mortality was assumed to estimate the excess mortality due to a positive test. Our findings align with evidence indicating an excess number of deaths in LTC homes across Ontario and Canada as well as internationally [4, 9, 16, 17, 27–31]. Ontario’s Long-Term Care COVID-19 Commission reported the province "was not prepared for a pandemic and that the province’s long-term care homes, which had been neglected for decades by successive governments, were easy targets for uncontrolled outbreaks" ([32] p2).

The stark difference in mortality observed in April 2020 and May 2020 coincides with Wave 1 of the pandemic in Ontario, during which there was insufficient personal preventive equipment for LTC staff and residents and no clear policies for LTC homes. For example, orders to limit the movement of LTC staff were not implemented until mid-April and were not initially applied to contract staff hired through employment agencies [33, 34]. When LTC homes managed to control the virus in June 2020, mortality rates for July to September decreased considerably and were slightly lower than the rates for the same period in 2019. For residents with positive COVID-19 tests, the drop in mortality for July to September is in keeping with other reports [9]. The decrease in mortality may be the result of various factors, including increased supports due to emergency funding from the provincial government and implementation of stricter rules in LTC homes [31]. Heightened screening, additional equipment, and containment strategies may have helped improve infection prevention and control in LTC homes during the summer months of 2020 [33]. The decrease in mortality could also be because the residents who survived Wave 1were younger or healthier at baseline. Yet the decrease was short lived as rates rose again in November 2020 and December 2020 during Wave 2.

The results from our study revealed differences in mortality based on key demographic and regional variations. In addition to variables such as age group and sex for which higher mortality rates are usually expected at different levels, the difference in mortality based on LTC home ownership provides critical evidence for early findings. We determined that mortality rates in privately owned homes were 25% higher compared to municipally owned homes. Stall and colleagues [31] found that for-profit status was associated with an increase in the extent of an outbreak and the number of resident deaths when compared to municipal and not-for-profit homes. However, they concluded that these differences were explained by a higher prevalence of older design standards in for-profit LTC homes and chain ownership.

The regional differences in excess mortality across LTC homes in Ontario suggest there may be gaps in the healthcare system that warrant attention from policymakers. Compared to Central Ontario, the test positivity rates for Eastern Ontario were almost half but the mortality rate was much higher. Research indicates that low population density within regions may be attributed to lower cumulative COVID-19 incidence rates in LTC homes [30, 31]. Our study demonstrates that even in areas with lower population densities additional factors may drive excess mortality. An international study of mortality in LTC homes determined that the share of resident deaths linked to COVID-19 was highly correlated to the number of COVID-19 deaths in the population that lived outside the homes [27].

### Limitations

There are several limitations to the data set used in our analysis. As with all administrative databases and issues related to the pandemic, this study is subject to coding error. The nature of the study means we cannot make predictions or infer causality; we can only report associations. Furthermore, death secondary to COVID-19 was inferred based on a test positivity rate. However, exact cause of death was not recorded in some of the data sets. Testing data may not have captured all residents with COVID-19, as mandatory testing in LTC homes was not implemented until well into Wave 1 of the pandemic.

## Conclusions

This study revealed important differences in mortality across years and positivity rates for residents of LTC homes in the largest Canadian province. Differences across regions and homes with various ownership models highlighted areas that require policy reform to improve resident care, especially during crises. Across the globe, countries have documented similar trends on the devastating effects of the pandemic on vulnerable populations living in long-term care homes. Findings from this study are an important contribution to the ongoing research about excess deaths due to COVID-19. The data provide evidence of the key factors that contributed to the increased number of deaths in long-term care. Future research is needed to identify the most relevant factors in explaining the differences between ownership models and regions as well as an analysis of excess mortality nationally.
